# “No data, no problem”? A critical reflection of health monitoring systems and equity during COVID-19 in Finland

**DOI:** 10.1186/s12913-026-14340-5

**Published:** 2026-03-11

**Authors:** Laura Kihlström, Markku Satokangas, Natalia Skogberg, Marjaana Viita-aho, Mika Gissler, Ilmo Keskimäki, Eeva Nykänen, Liina-Kaisa Tynkkynen

**Affiliations:** 1 Department of Healthcare and Social Welfare, Mannerheimintie 166, Helsinki, 00300 Finland; 2 Department of General Practice and Primary Health Care, University of Helsinki and Helsinki University Hospital, Tukholmankatu 8 B, Helsinki, 00290 Finland; 3 Department of Data and Analytics, Finnish Institute for Health and Welfare, Mannerheimintie 166, Helsinki, 00300 Finland; 4 Department of Molecular Medicine and Surgery, Karolinska Institutet, Stockholm, Sweden; 5 Faculty of Social Sciences, Tampere University, Kalevantie 4, Tampere, 33100 Finland; 6 Law School, University of Eastern Finland, Ylipistonkatu 2, Joensuu, Finland

**Keywords:** Data, Health monitoring, Health equity, Health systems and policy research, COVID-19

## Abstract

**Background:**

Lessons drawn from the COVID-19 pandemic have highlighted that health monitoring systems in various countries did not adequately capture how the pandemic affected individuals and groups in vulnerable situations. Disaggregated data on different population groups were often not available nor utilized to target responses. Much less attention, however, has been paid on why this was the case and what implications this may have had for health equity in both COVID-19 responses and future health system preparedness. We draw on health systems and policy research as well as social epidemiology to focus on data governance, i.e. the decisions, reasons, and factors affecting how data are collected, utilized, analyzed and presented in health systems.

**Methods:**

The article is written as a critical reflection in which we approach the Finnish response(s) to COVID-19 as a case study. We first describe the Finnish health monitoring system infrastructure prior to COVID-19, then present how data were utilized during the pandemic, and finally, demonstrate how data shaped responses, equity, as well as public perception of the crisis.

**Results:**

This critical reflection demonstrates how health monitoring systems, and particularly health system data, became both structural enablers and gatekeepers to equity during COVID-19 in Finland. A critical focus on data shows that the response in Finland during COVID-19 largely relied upon a homogenous perception of its population, reflected by the most commonly utilized data and indicators in national data dashboards. Disaggregated data for more tailored responses were eventually utilized, but only after the allocation of short-term additional resources.

**Conclusions:**

Based on our reflections regarding the COVID-19 pandemic in Finland, we argue that data have a critical role in framing health issues, garnering public attention for certain types of responses, and rendering certain population health issues visible or invisible. We discuss the implications of health monitoring and data practices in a single country context during COVID-19 for health system preparedness more generally. We conclude that equity-centered data and health monitoring practices should be viewed as a critical health system capacity.

## Introduction

Major shocks to health systems, such as pandemics, disproportionately affect individuals and communities in vulnerable situations. The COVID-19 outbreak was initially coined as the “great equalizer”. However, already early in the pandemic, it became astutely apparent that its impact was substantially more pronounced among groups that were at a greater social disadvantage already prior to COVID-19 [1–3]. This pattern is not an exception nor new. Indeed, country-level and global analyses completed during previous pandemics as well as COVID-19 demonstrate consistent socioeconomic, gendered, and racialized differences in the burden of disease due to a number of factors, such as lack of access to social safety nets and health care, poor quality of housing, pre-existing health conditions, and precarious working conditions [4–6]. These epidemiological patterns highlight the importance of health equity, the notion that systematic and consistent health disparities are unfair, avoidable, and remediable, as a crucial component of health system preparedness and governance of health system crises as they unfold [7, 8]. Promoting health equity during crises such as pandemics means putting the focus on reducing such disparities by actively taking into account the determinants that “adversely affect excluded or marginalized groups” [9].

Meaningfully detecting and measuring health disparities require systematic reporting, i.e. pre-planned collection of data with multiple individual characteristics that enable identification of marginalized groups to whom adverse outcomes may disproportionately accumulate [10, 11]. Such requirement for disaggregated data emphasizes the close linkage of how the information content of routinely-collected data – and the health monitoring systems in which they are generated – can shape our understanding of health equity [12–14, 11]. Disaggregated data pertains to the division of collected information into smaller units, and essentially involves categorizing the data into smaller groups to uncover disparities that might otherwise be obscured by existing data reporting methods. However, in many cases, disaggregated data are not available nor are they utilized effectively in national, regional, or local levels of health systems to promote equity-based approaches [15–18]. Therefore, data inequity, or the “continued invisibility and lack of representation in data for certain populations, particularly those with significant disparities” may hide and even exacerbate health inequities [13].

Data omissions and data gaps do not occur naturally but reflect the prioritization and decisions made by health systems actors themselves [11]. Health systems are social systems, and previous work in social epidemiology highlights that data are not neutral, but rather social products which are created, collected, and analyzed by agentic actors in health systems. Indeed, the phrase “no data, no problem” refers to how the act of collecting or not collecting data plays a crucial role in identifying and recognizing what problems public health sets out to solve [11]. Thus, given the central role that data play in public health, a critical focus on data in the context of health equity and health system crises is warranted. 

Crises and shocks, such as the COVID-19 pandemic, often make visible status quo structures and can thus also provide critical windows of opportunity for change and transformation [19–21]. There is a rich line of inquiry of analyzing various system shocks to understand the dynamics of politics and decision-making, and more recently there have been calls for investigating the power and politics of health system data, including a focus on how data are generated particularly during health system shocks [22]. For example, during COVID-19, the collection or non-collection of infection or mortality rates disaggregated by race, ethnicity, or socioeconomic status and other factors has been recognized as a key omission of surveillance and data presentation during the pandemic [22].While recognizing that this omission is critical, we find it important to further ask how, specifically, data shaped responses during COVID-19, what decisions and factors underlie such data practices, and how data practices may have influenced equity during the pandemic.

While posing such questions, we are also aware of the critiques against an overly data-oriented approach to health. Notions from critical data studies have pointed out how the datafication of health, epistemic governance by numbers, as well as the over-reliance on quantitative information in health sciences poses several challenges and problems [23–25]. For example, large data sets possess power as the act of categorizing people always reduces social difference and heterogeneity, and data on marginalized populations can in worst cases reproduce social harm and discrimination if the communities affected by such data are not involved in the process or if data are not adequately contextualized [14, 15, 26]. Cognizant of these critiques, we highlight that the use of data alone is no silver bullet to advancing health equity nor do better data necessarily lead to equity-focused decision-making [27].Collecting and utilizing disaggregated data should happen in tandem with careful ethical considerations, efforts which seek to advance long-term trust with different populations and communities, and with critical reflections on the power dynamics between researchers/research institutes and the populations studied [28].

Against this background, this article examines the Finnish response to COVID-19 from the viewpoint of health monitoring systems, data, and equity. The aftermath of the COVID-19 pandemic offers a valuable opportunity to critically assess the role of health monitoring systems and data during a global crisis. In the context of single-country case, we ask the question of how data shaped responses in a rapidly unfolding crisis, what factors underlie the health monitoring practices brought to the forefront by the COVID-19 pandemic, and how these may have impacted equity considerations during the pandemic. Finland’s response to COVID-19 has been highlighted as positive in international media, although several contested viewpoints have also been brought forward in research [29–31]. It can be stated that a comprehensive, critical evaluation and discussion of the Finnish COVID-19 response has not yet taken place nationally, perhaps as the pandemic was swiftly followed by a Russian unprovoked invasion in Ukraine, shifting the attention of European decision-makers from one crisis to the next [32]. Recognizing that we present a single country case for a wider international audience, we seek to contextualize the Finnish health system in detail in the article and place our findings in the broader context of health system organization and preparedness.

## Methods

The article is written as a critical reflection based on COVID-19 as a case study. Critical reflection has been widely employed in health professionals’ education and professional practice to critically assess practitioners’ assumptions and biases, including those affected by disciplinary, institutional, and societal systems, structures and ideologies, and to understand how such assumptions may influence practice [33]. While being commonplace in health professionals’ education, critical reflection has less so been utilized in assessing health system wide assumptions and power structures [34, 35]. By engaging in systems-level critical reflection, we seek to add to the growing field of inquiry for power-sensitive health systems and policy research, which urges scholars to assess how power shapes the research process itself, which requires reflection on how, why, and when evidence is (data are) gathered and through what kind of practices [28, 36–39]. Such a process of reflexivity is still only beginning to take shape in the broad field of health research which has traditionally relied upon large data sets and where health monitoring and data practices and rarely critically examined from the perspective of power and equity [14].

Building on the expertise of a multidisciplinary team of researchers with backgrounds in health sciences, health policy, law, medicine, epidemiology, information sciences, and medical anthropology, we synthesize and reflect upon health monitoring system features, real-life experiences and events during the COVID-19 pandemic, as well as our own published research pertaining to the governance and leadership of the pandemic. All authors of this article are affiliated with the Finnish Institute for Health Welfare (THL), which is a state research and expert institute that promotes the health, well-being and safety of the population. Taken together, the authors have decades of experience in health equity research and advocacy in Finland. COVID-19 was selected as a case study for the article as all authors have in some way or form been connected to the pandemic in their current positions, albeit not in roles which would include formulating policies and/or giving policy advice. Utilizing COVID-19 as a case study also allows to focus “on a single phenomenon within its real-life context” and helps to answer how and why certain health policy or system features exist [28]. Given that the subject matter of the article – health monitoring data and equity in the context of crises – is extensive, this article lays no claim on being in any way exhaustive.

The article structure is as follows. We first present a summary of the Finnish health system and its data infrastructure as context for our case study and reflection. Regarding the availability of disaggregated data as part of the Finnish data infrastructure, we focus more in depth on the availability of data pertaining to ethnic minorities and individuals with migrant backgrounds in Finland to highlight how certain populations have remained “hidden” in health monitoring systems. We then present elements of the Finnish national response to COVID-19 and describe the type of data which were predominantly used as a guide for decision-making, governance, and framing of the pandemic in Finland. We follow this with presenting how the utilization of disaggregated data helped create a community-based health promotion response during the pandemic. The case study section is followed by a reflection on factors that may have affected data collection and utilization practices, how these may have impacted equity, as well as how these may inform future health system preparedness.

### Case context

Finland is a high-income country with a universal health care system which in principle means that all permanent residents are entitled to health and social care services which are mainly funded by taxes and social security contributions. Parallel to the universal model, however, there exists other avenues for receiving care, including primary health care organized by employers through occupational health care schemes, as well as private services reimbursed through national health insurance and to a small extent financed also through voluntary private health insurances [40, 41]. Stark socioeconomic inequities are observed in nearly all areas of health and wellbeing, including in access to health services [42].

The routine collection of Finnish health and health care data also formed the backdrop to monitoring population health during COVID-19. Regarding such monitoring, the Nordic countries are often considered a “treasure trove of data”. Indeed, Finland has a long tradition of collecting various types of health and welfare data – a comprehensive overview of which can be found elsewhere [43]. These data can be used and disseminated for monitoring purposes and include population-based health interview surveys, health examination surveys and individual register data obtained either at local, regional, or national level. The different types of data complement each other. For example, national registers provide insight for usage of health care and social services, morbidity, and mortality, while population-based surveys provide insight into health behaviour and self-assessed health within population samples [43].

The commonly used Finnish registers in health services research have been collected for extensive time-periods and are generally considered of good quality [44, 45]. For example, the collection of nationwide inpatient data into the Care Register for Health Care (CRHC) began already in 1967, after which it has been further refined as well as supplemented with primary health care and specialized care outpatient consultations [45]. After its most recent addition of largest providers of private occupational health care services in 2019, CRHC now covers almost all health care use in entire Finland, even though reporting of visits to private providers remains still voluntary. Another example is the Finnish National Infectious Diseases Register, which contains communicable diseases notifications from 1989 onwards and thus also the number of COVID-19 tests, cases and deaths [43].

Moreover, unique personal identity codes introduced in the 1960s enable linkages in-between different health and welfare data as well as between these data and individual sociodemographic for more detailed analysis of population subgroups [46, 47]. However, while data protection regulations enable these linkages for statistical and research purposes, their usage for health monitoring, and policy purposes is more limited. The variables available in the Finnish registers are freely available in online data resource catalogues.Though compiled sociodemographic data available from Statistics Finland currently include several variables (such as age, sex, country of birth, parents’ country of birth, citizenship, language, marital status, family composition, education, labor market position, income, pensions, place of residence, and car ownership) some population groups are still poorly identified in the registers. For example, based on our experiences, compiling health data on ethnic minorities in Finland, often comes with multiple challenges regarding data linkage, lack of intersectional data, and lack of resources [17].

Independently of register data, development of population-based surveys has recently focused on better inclusion of ethnic minorities. For example, with the growing number of migrant origin persons in Finland, awareness on the need of disaggregated health data by migrant origin led to the launch of the Migrant Health and Wellbeing Study in 2010 [48]. Since then, several health and wellbeing population-based surveys sampling migrant origin populations have been conducted among working-age populations [49]. These surveys follow a similar study protocol and use comparable measures as surveys conducted among the general Finnish population. General population survey samples also include migrants but in the past the proportion of the total sampled participants was relatively small and would not have allowed disaggregation of data by country of birth or region of origin. Furthermore, migrant origin populations have in the past been less likely to take part in general population surveys due to language barriers. Surveys focusing on migrant origin populations had a weighted sample of the migrant origin population to ensure the possibility of disaggregation of data by region of origin and the most recent surveys have been offered in approximately 20 different languages. These surveys have also supplemented self-completed questionnaires with multilingual telephone interviews. In addition to surveys conducted among adults, the Finnish School Health Promotion Study, that is conducted biannually, also systematically produces statistics disaggregated by family, and more recently for example by gender identity [50].

In summary, the Finnish health monitoring system is broadly based on national surveys and register data from both national and local sources. Historically, data have been disaggregated by age group, sex, socioeconomic status, and region [43]. More recently, developments have been made to better capture the diversity of the population, including gathering health monitoring data or pinpointing to data gaps regarding ethnic minorities, sexual and gender minority populations, and undocumented immigrants. In other words, changes in the Finnish health monitoring system had been taking place years before COVID-19. Next, we turn to our case studies to summarize how data were utilized during the pandemic in Finland, and how they shaped the response(s) of the Finnish health system.

## Case study results

### The national COVID-19 response in Finland: data were reported at the aggregate level and affected the framing of the crisis

Pandemic preparedness and governance in Finland are influenced and steered by key regulatory frameworks. These include the National Pandemic Preparedness Plan, local and regional preparedness plans, and the Communicable Diseases Act [51]. Overall, these frameworks and documents place a high emphasis on the role of local and regional actors in pandemic governance, providing municipalities and regional state administrative agencies main decision-making powers in terms of non-pharmaceutical interventions and other restrictive measures (e.g. closing of schools and public premises). The Finnish Institute of Health and Welfare (THL) is responsible for maintaining national epidemiological surveillance systems in health system crises. This surveillance focuses on epidemiological variables such as number of cases, patients in hospital care, and mortality compiled into different registers.

The detailed chronological events related to the onset of the COVID-19 pandemic in Finland, as well as analyses of preparedness, leadership and governance of, and learning from the pandemic have been dealt with extensively in previously published articles by the authors [52, 53]. Overall, regarding governance, there were various shifts in centralization and decentralization of power during the first and second year of COVID-19, as has been the case in many European countries [52–54]. Most of the “surprises” and politicized aspects of governing the pandemic in Finland stemmed, according to our research, from tensions regarding roles and responsibilities. Tensions arose particularly during the acute crisis stage when predetermined frameworks for health system governance were not followed, particularly during the early months and the first year [52, 53]. Here, however, we focus on the role of data in how it both shaped and framed the Finnish national COVID-19 response.

From the beginning of COVID-19, starting from the acute stage unfolding in March 2020, and throughout its first year, certain key data were used as indicators of the severity of the situation. These data included number of COVID-19 cases in the population, data on intensive care unit and hospital capacity, number of COVID-19 related deaths, and later on, progress of COVID-19 vaccinations in the population [55]. These data were typically published in press conferences organized by the Prime Minister’s Office, Ministry for Social Affairs and Health, and THL, depending on the stage of the pandemic. These data were also publicly published on THL’s website, and the national media reported daily updates on the data up until the early months of 2022. These four main data sources were disaggregated by age, sex and region, however, such disaggregation was often not presented in national media, rather, the data were presented at the level of the entire population. Needless to say, these data also shaped public opinion and understanding of the pandemic.

In addition to these data, the public was also frequently informed about the classification regarding which populations were considered most at risk regarding COVID-19. These included individuals over the age of 70, as well as all individuals over the age of 12 who because of an illness or condition made them highly predisposed or predisposed for severe coronavirus disease. The latter category included individuals such as those waiting for organ transplantation or those who use anti-rejection medication due to stem cell transplantation, those undergoing active cancer treatment, those with Down syndrome, type 1 diabetes, morbid obesity, or psychosis, among others [56].

Based on our prior research completed with health system leaders in Finland, including politicians and civil servants, there were several conflicting viewpoints and tensions regarding the framing of the pandemic, as well as regarding how risk and vulnerability were defined. While the pandemic was a crisis which touched all sectors of society, our analyses suggest that it was still largely framed from the perspective of health system functioning and security, reflected by the most dominantly followed data and indicators. Disagreements existed particularly regarding whether or not the social and economic impact of measures to govern the pandemic could be considered sufficiently as the data focused intensively on the viewpoints of hospitals, particularly specialized health care [57].

It also become evident that there were only scant data available regarding populations in more precarious situations during COVID-19, such as undocumented migrants, and migrant workers. This, in turn, likely has had its impact on how preparedness plans and actions taken to mitigate the impacts of the pandemic could be tailored to population groups in different circumstances. According to our research, viewpoints from local and regional level civil servants also brought forward the need to track a wider variety of data and indicators at the national level, including unemployment rates, domestic violence cases, and child protection notices, as well as inclusion of the experiences of those utilizing social services [57]. It should be noted that parallel to this national response, there have also been smaller-scale responses. Next, we outline such a response focusing particularly on the needs of ethnic minorities in Finland.

### A tailored public health response for ethnic minorities during COVID-19 in Finland: the role of disaggregated data

In addition to the national response detailed in the prior section, there was also a smaller-scale, rapid COVID-19 response which focused on multilingual crisis communications tailored for ethnic minorities in Finland. THL monitored COVID-19 incidence and vaccine uptake by region of origin and mother tongue using data from the Infectious disease register and the Vaccine register, although these data were not reported as part of the national “data dashboard” described in the previous section. Additionally, THL monitored the perceived access to information and psychosocial and health impact of COVID-19 among migrant origin populations through a population-based MigCOVID Survey conducted during the first year of the pandemic [58]. Thus, disaggregated data were crucial to the smaller-scale response, as the data showed a higher incidence of COVID-19, and later lower vaccine coverage, among ethnic minorities in Finland. These data were utilized for tailoring and targeting multilingual crisis communications during the pandemic.

The need for multilingual crisis communications was astutely apparent already at the beginning of the COVID-19 pandemic [59]. Perceived access to information was lower among those who had lived in Finland for a shorter time and those who had at most intermediate Finnish or Swedish language skills, highlighting the need for multilingual and simple language crisis communications [60]. In response to the need for multilingual communications, a multilingual and multichannel COVID-19 task force was rapidly launched from the initiative of the Finnish Ministry for Social Affairs and Health. The coordination of the task force was positioned within THL as the responsible health authority for COVID-19 crisis communications to the general public. THL’s migration and cultural diversity team that runs research and development projects related to migration, cultural diversity, health, and wellbeing coordinated the multidisciplinary task force consisting also of experts from the communications department and COVID-19 response unit. Later on, also representatives from the Finnish Ministry of Economic Affairs and Employment, responsible for multilingual communications related to the economic impact of COVID-19, joined the multilingual crisis communications task force.

However, even though the multilingual multichannel COVID-19 communications task force was active from the beginning of the pandemic in Finland, and some basic written information on COVID-19 was produced in March in 20 different languages and in 13 languages by June 2020, it took until the fall 2020 that multilingual crisis communications were allocated sufficient resources to enable continuous production of key health messages multilingually and using the channels and format more accessible for linguistic minorities in Finland. Resources were allocated due to high numbers of COVID-19 cases, and later on in the epidemic due to lower vaccine uptake, among linguistic minority groups [60]. The allocation of extra resources was carried out across the Finnish health system during the COVID-19 pandemic [36]. With these additional funds, the multilingual crisis communications task force was able to produce regular up-to-date multilingual communications in various format, ranging from written instructions to infographics and video material. The languages were selected based on population statistics of the most commonly spoken languages in Finland [61]. Multilingual communications were also produced in the Saami and Roma languages, spoken by Finnish national minorities. Additionally, regularly updated information was published in six most commonly spoken foreign languages (Russian, Estonian, English, Somali, Sorani Kurdish, and Arabic). The number of languages had to be narrowed down because information changed rapidly, and the material production process was very time consuming.

In addition to creating multilingual information, substantial effort was put into effective dissemination of information. In addition to social media channels frequently used by the target populations, information was disseminated through radio channels in different languages, as well as question-and-answer sessions in different languages co-organized with collaborators from the non-governmental organizations (NGOs). Collaboration with NGOs was crucial during the pandemic both in reaching the target populations as well as in verifying the accuracy of the professionally translated multilingual communications. Partnership between THL and the Finnish Red Cross Multilingual and Multichannel Coronavirus Communications Coordination Project during 2021–2022 was also of key importance [62]. THL multilingual crisis communications task force also closely collaborated with key community representatives and regional health authorities. Collaboration was organized through roundtable discussions aiming at facilitating exchange of information and best practices. THL also ran social listening training for NGOs and regional health authorities, to support them in identifying information needs and most effective dissemination channels [63]. On a number of occasions, the task force ran a number of community dialogues with one linguistic group at a time. Such demand rouse for example when there were acute cases of misinformation or an acute spike in COVID-19 infections in certain linguistic groups or otherwise targeted and tailored measured were deemed as necessary.

## Discussion

### Why do data matter for health system responses and equity?

Given that the goal of public health is to maintain and improve the health of the entire population, diminishing unjust and remediable health disparities remains a key priority. This focus on equity is also justified by the fact that states have under international human rights conventions and their national constitutions an obligation to protect the right to health equity for different population groups [64]. The significance of statistical data in fields such as public health, epidemiology, and clinical medicine cannot be understated, as they are pivotal in monitoring population health patterns and in understanding the occurrences and management of diseases [65]. Data are powerful because they can shape policy, theories, value judgments as well as responses in health system crises. Disaggregated data provide a granular view of the impact of pandemics and other health crises on different demographic groups. By understanding the specific vulnerabilities and challenges faced by different groups, policymakers and public health officials can tailor interventions, allocate resources more effectively, and implement strategies that are more responsive to the diverse needs of communities.

In other words, health equity requires data equity, as data both shape notions and perceptions of health issues and pinpoint to existing inequities. Data equity entails that different population groups are adequately represented in health monitoring systems, so that inequities or disparities are not hidden [10, 12, 13, 66]. While lessons learned from the pandemic have focused particularly on the governance and leadership of COVID-19, in this article we have turned our gaze on data themselves. We have approached the Finnish health system monitoring data and the Finnish response(s) to COVID-19 as case studies to place health monitoring practices and their impact on crises responses at the forefront of our analysis.

### What can be stated about the role of data in shaping the Finnish COVID-19 response(s) and equity?

Our summaries of the Finnish response(s) to COVID-19 bring forward how data shape responses and equity during crises such as pandemics. First, the national data infrastructure for COVID-19 was set up relatively quickly, providing publicly available information on a daily basis, and speeding up health monitoring practices. The publicly available data on COVID-19 incidence, hospitalizations and deaths were crucial in garnering national attention and acceptance for certain political measures. However, the most commonly utilized data framed the pandemic from a largely biomedical perspective and mainly through aspects of health security and through its burden to the health care system, particularly specialized health care. This focus on data from specialized health care has been found to be a shared feature in European data dashboards and media discussions in the aftermath of the pandemic [66]. Second, parallel to the national-level response in Finland, a smaller-scale, targeted response was created to improve multi-lingual crisis communications to respond to the disproportionate impact that COVID-19 had on ethnic minorities in Finland. This effort was led by a team who had prior experience in the use of disaggregated data regarding minority populations, and therefore they had knowledge on how to include data from multiple sources, linking data from the Infectious Disease Register and Vaccine register (incidence of COVID-19, vaccine uptake) with data from the Population Register (age, sex, municipality of residence, country of birth, mother tongue, country of birth).

Finally, these findings bring forward that while the health monitoring system(s) were able to quickly produce publicly available data on the COVID-19 situation in Finland, there currently is no institutional, legal, or national mandate to set up disaggregated data infrastructure and health-monitoring systems which would allow for such data practices to be mainstreamed during health system crises. As a result, equity-centered health monitoring too often relies upon the motivation, knowledge, and funding available for individual researchers and teams who often have prior experience in minority or critical health equity research. Additionally, the data utilized at the national level did not provide granularity regarding how different social and structural factors (such as income, labor market status, housing situation, ethnicity, or immigration status) affected COVID-19 infections, deaths, and hospitalizations, which in turn also likely had an impact on how public health interventions were designed, implemented, and targeted at the national level. For example, publicly discussed risk factors for COVID-19 were broadly speaking based on age as well as medical conditions and did not include considerations of how risk might be amplified for those who might be structurally disadvantaged during COVID-19, such as by not being able to work remotely. Similarly, there was little public discussion or ethical considerations on how some COVID containment measures, such as lockdowns, may have impacted individual in assisted living units [67]. What this reveals is a lack of preparedness in the Finnish health monitoring system in acknowledging the diversity of the population as well as the various social, structural, and political issues which affect health outcomes and could be researched with data. In other words, these findings highlight how health monitoring systems and data can act as structural enablers or gatekeepers to equity during health system crises.

### What factors underpin data governance during COVID-19 in Finland?

We suggest that there are pragmatic, legislative, historical, and epistemological reasons behind the scant use of disaggregated data in the Finnish national response to COVID-19. From a pragmatic viewpoint, resources are often too few to utilize data to advance equity-based approaches. For example, while the Finnish health monitoring system during COVID-19 was set up relatively quickly, the targeted response which utilized disaggregated data had not been possible without additional funds which were dispatched from COVID-19 funds. Additionally, there are often lengthy permission processes which may delay the completion of register research involving disaggregated data. For example, while population background data are available for statistical purposes, and major health registers at THL can link data on birth country, language, nationality, education, socioeconomic status, and income as registered by Statistics Finland, these data cannot be used for health monitoring or scientific research without receiving study-specific permissions.

Similarly, for the collection of disaggregated survey data, a great deal of human resources and funding for equipment and services (translation, postal services, infrastructure etc.) are needed at the planning and implementation for disaggregated data collection. From past experiences in Finland, receiving an adequate response rate from migrants requires particular efforts including for example providing the opportunity to answer in the respondent’s mother tongue by translating the questionnaires/interviews; providing an opportunity to answer self-administered questionnaires both electronically and with paper and pen; and supplementing self-administered questionnaires with telephone interviews and/or door-to-door interviews with the help of trained multilingual interviewers. The translation process of questionnaires is lengthy, requiring several rounds of checks. A great deal of outreach work is needed to raise awareness on the importance to participate and build trust towards the survey. Additionally, the efforts of building the survey infrastructure are multiplied by the number of languages in which the survey is provided [68, 69].

Actions and decisions regarding data in health systems are likely also steeped in history and epistemology. Due to historical factors, the gathering of individual information categorized by specific characteristics has been a highly delicate matter in numerous European nations. For example, constitutional principles offer substantial protection for such sensitive personal information. Legislative frameworks such as the General Data Protection Regulation (GDPR), a data privacy and security law adopted in the European Union (EU) in 2016, places some restrictions on the use of disaggregated data in health system research. However, it should be noted that the GDPR does not prevent such research, rather, the European Commission, the main executive body of the EU, has called for research which utilizes disaggregated data (commonly referred to as “equality data”) [18]. In fact, the European Commission has published detailed instructions on ethical collection of disaggregated data and in the context of GDPR, placing emphasis on the doing no harm principle which posits that data collection should not reinforce existing discrimination, bias, or stereotypes, and that data collected “should be used for the benefit of the groups they describe and society as a whole” [70]. These instructions by the European Commission highlight that if disaggregated data are gathered in strict accordance with the established framework and its protective measures, data are recognized to be indispensable in combatting inequalities, inequities, social exclusion and discrimination [71].

The reasons behind non-collection or non-utilization of data can also be epistemological. We believe it to be possible that the ideals of universalism, egalitarianism, and equality – particularly the assumption that such ideals automatically lead to equity – may affect the scope of health system data and render some populations and health issues invisible even in universal systems. If a population is considered homogenous or uniform and as having equal opportunities, and if broader social structures are not conceptualized to affect health outcomes, decisions regarding data will reflect this [72]. In Finland, for example, considering the diversity of the population is a relatively recent phenomenon in health research, emerging only in the 21st century and increasingly during the last 10 years before COVID-19. How health issues – and health crises – affect and intersect among ethnic minorities, undocumented migrants, children of different backgrounds, older adults, sexual and gender minorities, single-headed households, or people with disabilities, for example, are still questions which often remain unanswered. In some cases, the lack of attention on use of disaggregated data may also result from considering topics on minority issues or social structures too sensitive, value-ridden, ethically problematic, or in general difficult to conceptualize in the field of health research. There is a critical need, including in Finland and elsewhere, for further training on conceptualizing health research from the viewpoint of social and structural injustices, particularly in the fields of epidemiology, medicine, and public health [11, 15, 16].

### How should findings on data during COVID-19 inform future health system preparedness?

The quest for data disaggregation is not new and precedes the pandemic. Particularly social scientists and critical health equity scholars have highlighted how data are central in identifying and addressing health inequities [11, 15, 16]. Additionally, at the European level, there have been union-wide calls for so called equality data to better understand how social problems, such as discrimination, affect health outcomes [18, 70]. The pandemic seems to have made visible several aspects of the Finnish health monitoring system, data gaps as well as potential for scaling up activities regarding the use of disaggregated data. As in other Nordic and European countries, we can conclude that Finland was unprepared in acknowledging the cultural, linguistic, and social diversity of the population in crisis preparedness and response measures prior to COVID-19 [73]. On the other hand, the findings demonstrate that when needed, and when allocated sufficient resources, health systems, particularly actors within the health systems, are also nimble in how they can be geared towards more equitable data practices.

There are several steps which can already be taken in anticipation of the next pandemic or another big crisis to which health systems also have to be prepared. Our findings indicate that equity considerations are easily sidelined during an acute crisis stage, and therefore health monitoring systems should be “geared” and set up to be more equity-focused as part of regular health system preparedness. Such preparedness activities could include the development of more detailed and ready-made sets of indicators for monitoring purposes that would focus on communities in vulnerable situations and capture health care and well-being from an equity perspective. For example, such an equity indicator could provide quantified data for example on whether different types of unwanted outcomes accumulate on some population groups – or whether these groups lack necessary services received by the population in general. During future crises, such indicators could provide a baseline for building further equity measures targeted specifically to the nature of such crises. Moreover, broadening the scope of indicators outside health care data (e.g., to social welfare) would better help decision-makers to acknowledge that the impacts of and decisions during health system crises are not limited only to health care.

Another important step would be to ensure that national legislations clearly authorize the building of equity indicators for monitoring purposes. For example, while Finnish legislation authorizes THL to monitor health system and describes the type of data THL can receive for such purposes, socioeconomic variables maintained by Statistics Finland are not included among the authorized data [74]. Nonetheless, THL receives these socioeconomic indicators for more strictly limited statistical purposes through the Statistics Act and through an agreement with Statistics Finland.^75^]. This enables long-term production of some equity indicators. However, all aggregated statistics are required to be published regularly, which limits the production of such indicators to those that do not risk stigmatizing groups in vulnerable situations. In other words, though Finland aims to advance health and health care equity, authorizes national health system monitoring, and collects high-quality individual data, its legislation provides only limited support for building tools for monitor health and health care equity. Future research is needed to account for these institutional challenges as they pertain to equitable health monitoring systems.

Lessons learned from COVID-19 hopefully are turned into new type of thinking around health system preparedness. We agree with colleagues in health systems and policy research that instead of technically gearing up for the next “new” COVID-19, more efforts should be paid on “general preparedness” which includes rehearsals, clarification of roles and responsibilities, and investments in information systems [76]. We have argued throughout this article that health monitoring practices, i.e. information systems, are at the heart of how health systems can strive for health equity, as they can be used to frame health issues, garner public attention for certain types of responses, and render certain population health issues visible or invisible. Indeed, data practices which focus on equity by acknowledging the needs and realities of different population groups are a critical system capability and should be highlighted as such.

### Limitations

There are limitations to our article. The purpose of this critical reflection has been to reflect upon and understand how decisions made by health system actors themselves shape responses and frame health issues based on the type of data brought forward and analyzed. We encourage researchers and professionals in similar fields in other countries to regularly assess their health monitoring practices, in their unique legal-political contexts, communities, and cultures. This work will require joint expertise from public health, social sciences, data sciences, as well as policy and law. Such work is necessary to ensure that health equity is more than just an afterthought in crises that health systems inevitable will face in the future as well. However, there are also downsides to engaging in self-assessment of one’s own work, especially since all authors are currently employed at THL, a national health institute. This critical reflection therefore privileges the viewpoints of those with more power in the health system, and should be complemented by further critiques and analyses, especially by listening to those who have been targeted by COVID-19 policies and responses.

While our article focused on disaggregated data pertaining ethnic minorities, there are obviously other data gaps which should and can be addressed by health monitoring systems. Research is also needed on how the use of non-sensitive proxy indicators, such as work sector, could be utilized as a source of information during rapidly unfolding crises. Furthermore, it is likely that internationally reported undercounting of COVID-19 events, hospitalisations and mortality exist also in Finland, especially in the early stages of the pandemic (during the build-up of testing capacity) as well as when COVID restrictions were lifted (due to dismantling of regular testing and regulations before 80% vaccine coverage in the population)^77^]. Though the coverage of causes of death statistics in Finland is of good quality [44], it is still possible that number of untested COVID deaths may have been misclassified under other conditions, for example dementia or cardiovascular causes [78]. Testing also varied regionally during the pandemic which may have led to respective variations in identified COVID cases [79]. It is possible that such variations may include further data inequities. However, because of well-established monitoring system in hospitals, the coverage of patients with severe COVID treated in inpatient care or intensive care units is of good-quality in register data [45].

## Conclusion

Because pandemics and other health system shocks often disproportionately affect individuals and communities in vulnerable positions, health equity should be a key component of health system preparedness and governance. Health equity requires data equity, i.e. sufficiently disaggregated data to document, monitor, and address unjust and remediable health disparities, and therefore critical evaluations should be made about health monitoring systems during COVID-19. In this article, we have engaged in critical reflection to examine the Finnish health monitoring system and how its data has been utilized during COVID-19. While the Finnish health system is known for its rich registry data, population-based health surveys, and universal health care system, this article points out several understudied areas of health inequities which are currently not reflected in Finnish data collection and utilization processes. We have discussed how data shaped COVID-19 responses in Finland, and what pragmatic, legislative, historical, ethical, and epistemological factors underpin current health monitoring practices. These observations highlight that all health systems – regardless of how they are organized – need to do better in addressing the diverse needs of individuals and communities. Concentrated efforts are needed to center equity in health monitoring systems, and positive experiences as well as challenges made visible during COVID-19 should be utilized to inform and scale up equity-centered data collection, utilization, and dissemination practices.

## Data Availability

No datasets were generated or analysed during the current study.
